# The Special Issue: Transmission and Detection of Food and Environmental Pathogens

**DOI:** 10.3390/microorganisms9122611

**Published:** 2021-12-17

**Authors:** Nigel Cook

**Affiliations:** Jorvik Food Safety Services, York YO32 2GN, UK; nigelcook@foodsafetyteam.org

To progress towards a full comprehension of the risk caused by pathogenic microorganisms transmitted via food and environmental routes, extensive information on the prevalence, the mechanisms of contamination, and the survival of pathogens is required, particularly to assist with the development of effective systems to reduce contamination, and to formulate procedural control measures, such as the implementation of food safety criteria. The Special Issue, “Transmission and Detection of Food and Environmental Pathogens”, of *Microorganisms* presents several facets of research, which should facilitate further acquisition of information, particularly into the prevalence and transmission routes of food and environmentally transmitted microbial pathogens.

The occurrence of microorganisms resistant to antimicrobials and their potential introduction into the food supply chain is of continuing concern. The study by Ge et al. [1] examined the prevalence of antibiotic resistances among two bacteria commonly used as indicator organisms for potential pathogen presence in animal feedstuffs. An extensive survey of several product types was performed, and each indicator isolated from a significant proportion of samples. The resistance to one or more antibiotics was observed in several isolates. Although resistance rates were lower in animal foods than previously observed in retail meat, the presence of antimicrobial resistance in materials at the very beginning of the animal food product supply chain prompts the need for the consideration of routine monitoring of feedstuffs for resistant microorganisms.

International standard methods exist to enable the monitoring of a range of pathogenic microorganisms. Although effective, many can benefit from regular updating and refinement. Kubina et al. [2] modified an existing standard for the detection of the protozoan parasite *Cryptosporidium parvum* on leafy green vegetables, to incorporate a real-time PCR assay harnessed to cell culture. The modified method will be a useful tool to facilitate the detection and quantitation of infectious *C. parvum* on fresh produce, and can potentially be incorporated into future standards.

The Special Issue includes three studies covering the prevalence and survival of *Listeria* in food production settings. By examining some common conditions found in such settings, Manso et al. [3] found that oxidative stress (e.g., during disinfection procedures) at refrigeration temperatures could result in the increased expression of certain virulence genes in *L. monocytogenes*, thus increasing the risk of virulent strains of this bacterium during food production. Rossi et al. [4] analysed, by PCR and other molecular techniques, the characteristics of *Listeria* species isolated from various food and food preparation surface samples collected in Northern Italy. They found virulence factors commonly associated with *L. monocytogenes* in other, generally innocuous, *Listeria* species. The potential virulence of these other species indicates a need for expansion of food control procedures to include all *Listeria* species. Kaszoni-Rückerl et al. [5], reporting the persistence of *L. innocua* in food production environments extensively colonised by *L. monocytogenes*, with the former bacterium being found to have similarities in genetic markers with the latter, discussed the requirement for further research into the genetic exchange between *Listeria* species, to inform the design of targeted food safety measures.

Reflecting on the need for the effective implementation of food safety assurance systems in wet markets, Ngan et al. [6] performed a bacterial profiling study on cutting boards used for processing pork, poultry, and seafood products in these settings, in Hong Kong. A range of species, some pathogenic, was identified, with boards used for pork products harbouring the most types. The boards from traditional wet markets had a higher abundance and diversity of species compared to those from modern wet markets, as did, not unexpectedly, boards which were not cleaned compared to those that were. The most common cleaning method was the scraping of boards without additional disinfection; while the study found that this did reduce the microbial load, it was not fully effective in removing all bacterial types, which therefore had the potential for the contamination of foodstuffs.

A very common approach to cleaning food preparation surfaces in domestic settings is the use of kitchen sponges. However, if these become contaminated with microorganisms they can transfer them, through cross-contamination, to utensils and surfaces. Considering the previous information that the regular disinfection of sponges might not be wholly effective in removing microbial contamination, Jacksch et al. [7] examined untreated and regularly microwaved sponges, using metagenomic sequencing. They found an abundance of microbial species, including bacteriophages, on the untreated sponges. Regular microwaving appeared to promote the increase in biofilm-forming bacteria, such as *Acinetobacter* and *Pseudomonas*. The analysis also disclosed a higher abundance of genes required for sulphur metabolism. The authors recommended that these findings be followed up to ascertain any impact on domestic hygiene and human health.

This Special Issue has provided new insights into the persistence of a variety of microbial types in food settings. It is hoped that the leading-edge data and information it contains will promote further progress in the detection and characterisation of food and environmentally-transmitted microorganisms.
